# Analysis of the Intestinal Flora in Male Versus Female Swamp Eels (*Monopterus albus*)

**DOI:** 10.3389/fmicb.2020.00699

**Published:** 2020-04-30

**Authors:** Ying Wang, Jinhua Zhang, Qiubai Zhou, Zirui Wang, Miao Gao, Xin Yang, Yu Liu, Zhengzhou Zhang, Wenhao Jiang, Chonghua Hu, Wenping Zhang

**Affiliations:** Aquatic Animal Research Laboratory, College of Animal Science and Technology, Jiangxi Agricultural University, Nanchang, China

**Keywords:** swamp eel, high-throughput sequencing, intestinal flora, sex reversal, sex differences

## Abstract

This study aimed to analyze the intestinal flora of male versus female swamp eels, which have the unique characteristic of sex reversal. Same-aged swamp eels from the same parents, kept under the same conditions in terms of environment, diet, etc., were used as the study materials. After breeding for 1 year, 18 healthy swamp eels (nine males and nine females), weighing 39.4 ± 2.3 g, were selected. The intestinal contents of three swamp eels of the same sex were combined and labeled as follows: FM1-FM3 (*n* = 3) for females and MM1-MM3 (*n* = 3) for males. High-throughput sequencing was used to research the v3-v4 region of 16S rDNA in the intestinal flora. The results suggested significant differences in the structure, composition, and diversity of the intestinal flora of male versus female swamp eels. The relative abundances of Actinobacteria and Spirochaetes in female swamp eels were significantly higher (*p* < 0.05) than in male swamp eels at the phylum level. The relative abundances of *Mycobacterium*, *Bacillus*, and *Vagococcus* in female swamp eels were considerably higher (*p* < 0.05) than in male swamp eels at the genus level. The Alpha diversity of females was higher than that of males, and the Shannon index of females was also significantly higher (*p* < 0.05, Tukey’s HSD) than that in males. Investigations of Beta diversity, including NMDS ordination, UPGMA cluster analysis, and PLS-DA analysis, showed that female and male swamp eels could be clearly distinguished based on significant differences in intestinal flora between the FM group and the MM group. LEfSe analysis showed that the dominant bacteria were *Cetobacterium* in male swamp eels and *Clostridium_sp_ND2*, *Mycobacterium*, *Alphaproteobacteria*, and *Bacilli* in female swamp eels. The results showed dramatic differences in the intestinal flora between the sexes, which indicates the need for a more in-depth study on sex reversal in the future.

## Introduction

The swamp eel (*Monopterus albus*) is rich in nutrition, has very palatable meat, and is favored by consumers. It is a major freshwater economic fish in China (Chi et al., 2017). It has the characteristic of undergoing a sex reversal: during the embryonic stage and to the first sexual maturity, it is female; it then enters an intersex state, and then some individuals develop into males (Zhao et al., 2018). The number of wild swamp eels is steadily decreasing with increasing demand in domestic and foreign markets. Large-scale artificial breeding has become an inevitable trend. However, because swamp eel is initially female and then transforms into a male, the size of the female swamp eel is small, and the number of eggs is low. This limits the large-scale production of swamp eels. If the sex reversal of swamp eels can be regulated, it may provide an effective way to facilitate their large-scale breeding. Thus, numerous studies have been conducted from the perspective of genes and epigenetics, which have shown some promising results (Zhou et al., 2002; Wang et al., 2003). However, the sex reversal of swamp eel has not been reported from the perspective of intestinal flora.

The intestinal flora is considered an integral part of the organism (Bi et al., 2015). Also, the intestinal flora has been regarded as a large and complex ecosystem in the body, which is affected by diet, physiological status, environmental factors, genetic factors, and other factors (Filippo et al., 2010; O’Toole, 2012; Zijlmans et al., 2015; Karl et al., 2017). The intestinal flora of fish is also affected by many factors, including diet, developmental stage, environmental temperature, and growth rate (Maiwore et al., 2009; Campbell and Buswell, 2010; Ringø et al., 2016; Neuman et al., 2016; Lin et al., 2019). However, the impact of sex on intestinal flora is unclear. Studies have shown that the feeding habits of *Boleophthalmus pectinirostris* and *Periophthalmus magnuspinnatus* have more significant effects on the intestinal flora than sex (Ma et al., 2018). However, under the same feeding habits, significant differences were found in the intestinal flora of males versus females (Ma et al., 2018). A significant correlation between sex and intestinal flora in *Gasterosteus aculeatus* and *Perca fluviatilis* was also reported (Bolnick et al., 2014; Ma et al., 2018). These studies suggested that intestinal flora might be significantly associated with sex in fish.

This study elucidates the basic composition of intestinal flora in swamp eels and identifies the key bacteria in male versus female swamp eels to provide useful information for the study of the sex reversal mechanism in male and female swamp eels.

## Materials and Methods

### Sample Collection

With the approval of the animal care and use committee of Jiangxi Agricultural University, the study was conducted out in the aquaculture base of Jiangxi Agricultural University (28°45′N,115°49′E), Jiangxi, China. At the start of the experiment, the swamp eels were cultured from eggs to 4 months of age, and 162 fry of the same size and strong physique, with an average body weight of 5.70 ± 0.05 g, were screened from all the fry in the study. The swamp eels were put into a cage (1.0 × 2.0 × 1.0 m), and the cage was placed in a 3.5 × 1.2 × 1.2 m cement pool. To simulate the actual culture environment, 95% freshwater hyacinth (*Eichhornia crassipes*) was placed in the cage. The bottom of the pool had the shape of a pot bottom. A drainage hole with a diameter of 110 mm was made in the lowest part of the pool. A water inlet hole with a diameter of 30 mm was made in the upper part of the pool. The inlet was controlled by a valve to adjust the volume of water flow. Through air lift by a fan, the flow of water could be promoted and the amount of oxygen could be increased. The temperature of the aquaculture water was 25–28°C, the pH value was 7.2–8.0, and the dissolved oxygen was >5 mg/L. The experimental diets are listed in Supplementary Table S1. Fish were fed with the same experimental feed twice a day (08:00 and 18:00). Eighteen swamp eels (nine males and nine females) of the same size and strong physique, with an average body weight of 39.4 ± 2.3 g, were selected from 162 swamp eels and cultured for 8 months. Swamp eels were transported to the laboratory over ice and anesthetized with 60 mg/l^–1^ doses of MS-222 for 10 min. The fish body surface was then swabbed with 75% ethanol two to three times. After sterile dissection, the intestinal contents were taken into a sterile microcentrifuge tube, put into liquid nitrogen for quick freezing, and transferred to an ultra-low-temperature refrigerator at −80°C for preservation. Each intestinal content replicate was pooled from three sampled fish (same gender) and labeled as follows: FM1-FM3 (*n* = 3) for female swamp eels and MM1-MM3 (*n* = 3) for male swamp eels.

### DNA Extraction, PCR Amplification, and Illumina Sequencing

Intestinal microbial DNA was extracted by the DNA extraction kit of Tiangen Biochemical Technology Co., Ltd. (Beijing). The DNA samples that passed the quality test were sent to Major Bio-Technology Co., Ltd., for amplification and sequencing. After the v3-v4 region of 16S rDNA was amplified by fusing primers with 338F and 806R barcodes, an Illumina MiSeq platform was used for high-throughput sequencing (Han et al., 2019). According to barcode and primer sequence, all raw data were screened by using Quantitative Insight into Microbial Ecology (QIIME) (Zheng et al., 2017; Han et al., 2019).

### Bio-Informational Analysis

Some of the raw data obtained by sequence were useless data. To make the results of information analysis more accurate, the raw data were spliced and filtered to obtain effective data (Bokulich et al., 2013; Xing et al., 2019). Based on valid data, the operational taxonomic units (OTUs) were clustered by USEARCH (version 7.0)^1^ with 97% identity as the division standard (Han et al., 2019). The representative sequence in each OTU was screened and used to annotate taxonomic information using the Silva Database^2^ based on the Mothur algorithm. Alpha diversity statistics for each sample were calculated by Mothur (version 1.30.1), including the observed richness (Sobs), the Simpson diversity index, the Shannon diversity index, the Chao1 richness estimator, the ACE estimator, the phylogenetic diversity (PD), and Good’s coverage. Beta diversity assessment included non-metric multidimensional scaling analysis (NMDS), a cluster analysis diagram based on the Unweighted Pair-group Method with Arithmetic Mean (UPGMA), and Partial Least Squares Discriminant Analysis (PLS-DA). NMDS ordination can observe the degree of difference between samples based on the Binary_Euclidean distance of OTUs. Bacterial taxonomic differences between the FM group and the MM group at the genus or other taxonomic level were analyzed using LEfSe. SPSS 22.0 was used to perform statistical analysis. The sequence information for this study has been uploaded to GenBank with the accession number SRP230335.

## Results

### 16S rDNA Gene Sequencing and Alpha Diversity

In total, 266441 good quality V3-V4 regions of 16S rDNA sequences were detected from the six samples. Each sample was resampled to the minimum number of sample sequences (39549 reads per sample) and clustered, resulting in 407 OTUs of 97% identity, with the number of OTUs in each sample ranging from 67 to 310. In order to evaluate the depth of sequencing and species richness, a rarefaction curve was constructed for each sample. The rarefaction curves (Supplementary Figure S1) showed that most of the OTUs in all samples were detected and that all samples reached saturation. At the same time, the Good’s coverage indices of all samples were as high as 99%, indicating that the depth of sequencing was sufficient (Table 1). The Alpha diversity of intestinal flora was calculated by the Sobs, Chao1, ACE, PD-whole-tree, Shannon, and Simpson diversity indices. The specific results on Alpha diversity are shown in Table 1. The Alpha diversity of females was higher than that of males, and the Shannon index of females was also significantly higher (*p* < 0.05, Tukey’s HSD) than that in males. The results suggested that sex had a positive influence on the species diversity of intestinal flora in swamp eels.

**TABLE 1:** Differences in intestinal microbial alpha diversity in swamp eels of different sex.

**Item**	**MM**	**FM**	***P*-value**
Sobs	93.667 ± 19.86	225.33 ± 83.80	0.106
Shannon	0.82 ± 0.28	1.72 ± 0.11	0.020*
Simpson	0.66 ± 0.17	0.39 ± 0.08	0.100
Chao1	139.34 ± 43.08	253.03 ± 99.47	0.176
ACE	173.51 ± 69.10	255.44 ± 83.58	0.263
PD-whole-tree	12.68 ± 1.84	21.33 ± 5.97	0.119
Coverage	0.99 ± 0.00	0.99 ± 0.00	0.445

The data are expressed as the mean values ± standard deviation (SD). *P*-values were determined using Welch’s *t*-test (*P < 0.05).

### Composition of Intestinal Microflora in Female and Male Swamp Eels

**FIGURE 1:**
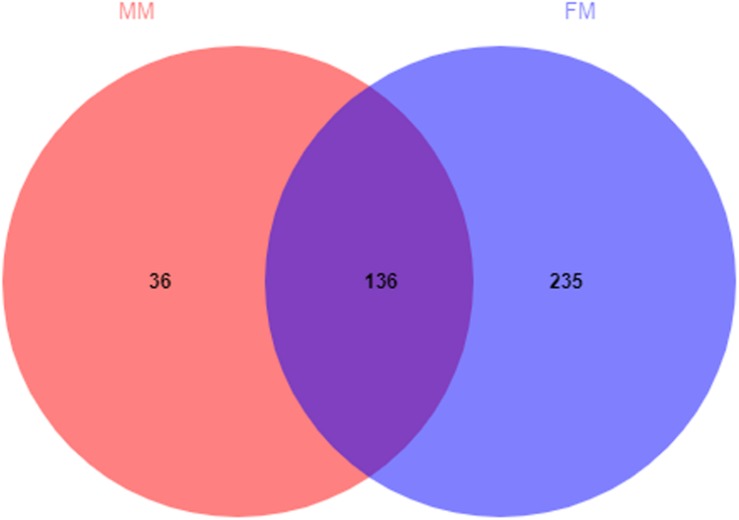
Venn diagram of OTUs in the female and the male swamp eels. The numbers of shared and unique OTUs are displayed.

**FIGURE 2:**
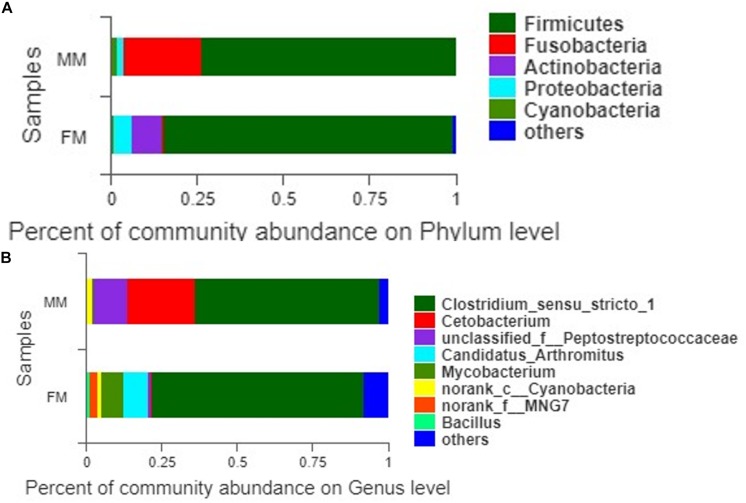
The intestinal bacterial communities at the phylum level **(A)** and the genus level **(B)**.

A total of 407 OTUs was obtained in all samples, of which 136 OTUs were shared between the female and male swamp eel samples. The numbers of unique OTUs in the female and male swamp eel samples were 235 and 36, respectively (Figure 1). According to the results of compositional analysis, a total of 12 different phyla was identified in all samples. After merging areas constituting less than 1%, all samples were found to be concentrated in five phyla (Firmicutes, Fusobacteria, Proteobacteria, Actinobacteria, and cyanobacteria) (Figure 2A). In the FM group, the dominant phyla were Firmicutes (84.23%), Actinobacteria (8.46%), and Proteobacteria (5.49%). In the MM group, the dominant phyla were Firmicutes (73.43%) and Fusobacteria (22.55%). From the relationship between the sample and the species (Supplementary Figure S2), the Firmicutes phylum was distributed in the two groups, and the distribution ratio (FM:53%, MM:47%) was similar. Actinobacteria (97%) and Proteobacteria (78%) were mainly distributed in the FM group, while Fusobacteria (98%) were mainly distributed in the MM group. These results suggest that the predominant phylum of the two groups was the same but that the other dominant phylum was different. Next, we analyzed the comparative composition of intestinal flora in the two groups at the genus level. After merging areas constituting less than 1%, all samples were found to be concentrated in eight genera

(Figure 2B). In the FM group, the dominant genera were *Clostridium_sensu_stricto_1* (70.11%), *Candidatus_Arthromitus* (8.35%), *Mycobacterium* (7.33%), *norank_f_MNG7* (2.82%), and *Bacillus* (1.22%). In the MM group, the dominant genera were *Clostridium_sensu_stricto_1* (61.12%), *Cetobacterium* (22.55%), unclassified_f_Peptostreptococcaceae (11.37%), and norank_c_Cyanobacteria (2.07%).

### Beta Diversity of Intestinal Microflora in Female and Male Swamp Eels

**FIGURE 3:**
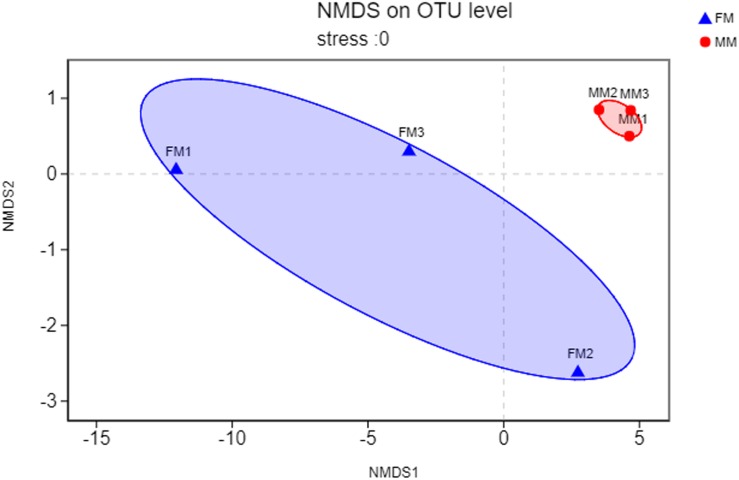
NMDS ordination based on Binary_euclidean similarities of bacterial communities. The blue triangles represent female swamp eels (FM), and the red circles represent male swamp eels (MM).

**FIGURE 4:**
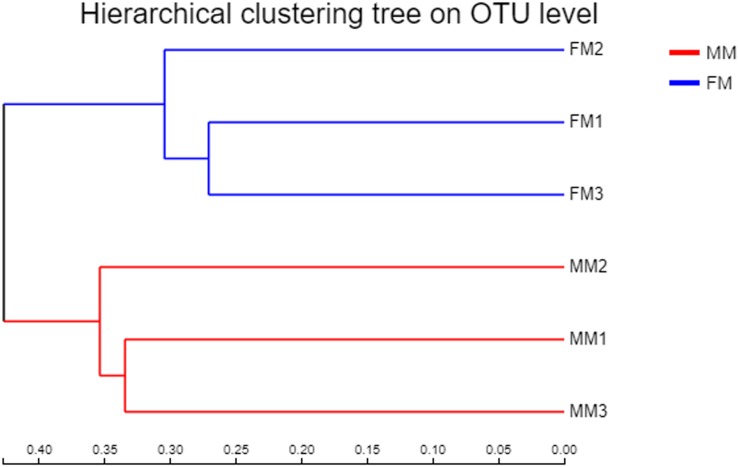
Clustering tree constructed by UPGMA (FM, female swamp eel; MM, male swamp eel).

Non-metric multidimensional scaling analysis ordination based on the OTU abundance of each swamp eel sample was used to show the relationship between the FM group and the MM group more clearly. The NMDS ordination results showed that the three male samples were very close to each other and could be separated from the three female samples, which indicated significant differences in community structure between male and female swamp eels (Figure 3). Meanwhile, the cluster analysis diagram based on UPGMA showed similar results, wherein samples of FM1, FM2, and FM3 were clustered into one branch, while samples of MM1, MM2, and MM3 were clustered into another, which illustrated that the difference between samples was mainly due to sex (Figure 4). The PLS-DA analysis results showed that the female and male samples could be distinguished and grouped into two groups, indicating that the intestinal flora compositions of female and male eels were significantly different. Besides, the dispersion of the sample point distribution in PLS-DA analysis showed major variations in the composition of the intestinal flora of different female samples (Supplementary Figure S3).

### Difference in Microbial Composition in Female Versus Male Swamp Eels

**FIGURE 5:**
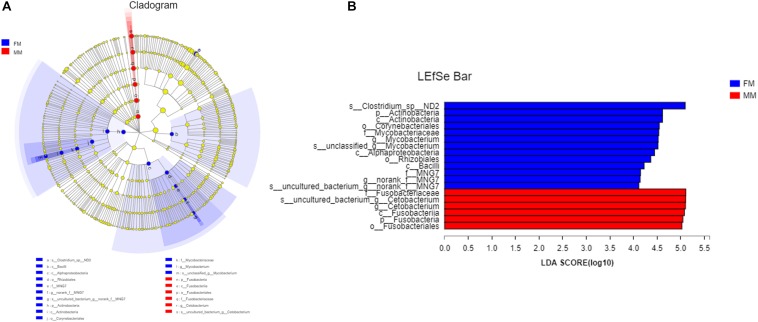
LEfSe map of species differences between the FM group and MM group at the species level. **(A)** LEfSe cladogram, **(B)** highly significant bacteria with LDA score >4.

Analysis of the differences between the two groups showed a significant difference (*p* < 0.05) in Actinobacteria and Saccharibacteria between the FM group and MM group, but there was no significant difference in other phyla (*p* > 0.05) (Supplementary Figure S4). At the genus level, the relative abundances of *Mycobacterium*, *Bacillus*, and *Vagococcus* in the FM group were significantly higher than those in the MM group (*p* < 0.05) (Supplementary Figure S5). At the species level (>1%), *Candidatus_Arthromitus* only existed in the FM group, while *Porphyromonadaceae, Halanaerobium, Lachnospiraceae_bacterium_mt14, Dechlo romonas, Lysobacter_ximonensis, Chryseobacterium, Aeromonas_sharmana, Duganella*, and *Nitrospira* only existed in the MM group (Supplementary Figures S6, S7). As shown in Figure 1, the FM group had more species than the MM group. To more accurately identify biomarkers between the two groups, LEfSe analysis was used to identify the key roles with significant effects in the FM group and the MM group. The threshold for the LDA score was set to 4.0. The LEfSe cladogram (Figure 5A) showed that Actinobacteria and Fusobacteria were divided between the MM and FM groups: Actinobacteria in the FM group, and Fusobacteria in the MM group. In contrast to female swamp eels, the genus *Cetobacterium* (LDA score = 5.100, *P* = 0.049) and the phylum Fusobacteria (LDA score = 4.066, *p* = 0.024) were predominant in the intestinal flora of the male eel. Meanwhile, the species *Clostridium_sp_ND2* (LDA score = 5.096, *p* = 0.049), the genus Mycobacterium (LDA score = 5.220, *p* = 0.049), the class Alphaproteobacteria (LDA score = 4.444, *p* = 0.049), and *bacilli* (LDA score = 4.226, *p* = 0.049) were predominant in female swamp eels (Figure 5B). When the threshold was adjusted to 2.0, numerous differential dominant species appeared in females but not in males (Supplementary Figure S8).

## Discussion

In the early stage of fish growth and development, the intestinal flora mainly comes from diet, the surrounding water, and the soil environment, and it changes dynamically with the growth and development of fish, Li et al. (2014), Stephens et al. (2016), and Ma et al. (2018). Some studies have pointed out that different sexes of fish display different physiological characteristics and behaviors during reproduction (Liao et al., 2014; Ma et al., 2018). The differences in intestinal flora between male and female swamp eels have not been studied. Factors such as diet, host genetic background, and age can influence intestinal flora in the same host with different sex (Org et al., 2016). Hence, the swamp eels in this experiment were fed the same experimental diet and kept in the same aquatic environment to ensure that the conditions were the same for all eels. Sex was considered as the only influencing factor when determining the intestinal flora of swamp eels of different sex by high-throughput sequencing. The differences in intestinal flora of swamp eels of different sex were analyzed and compared. The Shannon index in the female swamp eels was higher (*p* < 0.05) than that in males. There was no significant difference in other Alpha diversity indexes, but the diversity and richness of the female swamp eels were higher than in the males. The Alpha diversity of males was higher than that of females in zebrafish and *B. pectinirostris* (Liu et al., 2016; Ma et al., 2018), which was contrary to the results of this study. This may be caused by species differences or the unique sex reversal in swamp eels, which accounts for the diversity of intestinal flora in swamp eels compared to common fish. There are few relevant studies and insufficient information, so the specific mechanism requires further study. Meanwhile, the Beta diversity analysis found significant differences in intestinal microflora between female and male swamp eels. The distribution of the male sample was clustered, while the female sample was scattered. This indicates that the female has higher specificity in the intestinal flora, suggesting that the intestinal flora of swamp eels changes at different developmental stages.

The intestinal flora in swamp eel was previously reported to be mainly Firmicutes, Fusobacteria, and Proteobacteria (Peng et al., 2019), which was similar to the results of this study. Analysis of the composition of intestinal flora in female versus male swamp eels showed that Actinobacteria content was significantly higher (*P* < 0.05) in females than in males at the phylum level. Fusobacteria were more abundant in males than in females. Studies in zebrafish and *B. pectinirostris* showed that the abundance of Fusobacteria in males was lower than that in females (Liu et al., 2016; Ma et al., 2018), which was contrary to the results of this study. However, studies on mice showed that the abundance of Actinobacteria in females was higher than that in females (Org et al., 2016), which was consistent with this study. At the phylum level, the results of very distant host species (swamp eel and mice) are similar, while the results of closer host species (swamp eel, zebrafish, and *B. pectinirostris*) are the opposite, for unknown reasons. The sex reversal of swamp eel could perhaps lead to the diversity of intestinal microbes, which differed from that of regular fish. This hypothesis remains to be tested. There are several differences in physiological structure and lifestyle between swamp eels and mice. We further compared them at the genus and the species levels, which showed substantial differences. Therefore, although the two are similar at the phylum level, the microorganisms that can survive and colonize their intestines are very different. This study found that the higher abundance of Fusobacteria in male swamp eels was due to the higher abundance of *Cetobacterium*. *Cetobacterium* is the core flora in the intestinal tract of fish (Jiajia et al., 2012). It can produce acetic acid to promote protein synthesis or combine with coenzyme A to boost sugar and fat metabolism and can synthesize fats, carbohydrates, and proteins for the host’s use (Lin et al., 2019). This suggests that *Cetobacterium* may play a vital role in the development and growth of fish. The abundance of *Cetobacterium* in the intestine of male swamp eels was 22.55%, and that in female swamp eels was 0.46%. The LEfSe analysis results showed that *Cetobacterium* was the differentially predominant species in male swamp eels. We speculate that it may play a key role in the sex reversal of swamp eels. The two groups of dominant symbiotic bacteria are *Clostridium_sensu_stricto_1*. It has the highest content in the healthy growth population and was the major member of intestinal bacteria in some fish (Sullam et al., 2012; Lopetuso et al., 2013; Lin et al., 2019). The abundance of *Clostridium_sensu_stricto_1* in the intestine of healthy piglets is higher, and decrease in its abundance may cause diarrhea in healthy piglets (Han et al., 2019). This suggests that *Clostridium_sensu_stricto_1* plays an essential role in maintaining intestinal homeostasis. LEfSe analysis showed that *Clostridium_sp_ND2*, *Mycobacterium*, *Alphaproteobacteria*, and *Bacilli* are the dominant genus in female swamp eels, and the sex dependence of these bacteria may be related to the sex reversal of swamp eels. It is speculated that their existence may have a specific regulatory effect on the sex reversal of swamp eels. There are no reports on Clostridium_sp_ND2. *Mycobacteria* are commonly found in humans and animals with infectious diseases, so they are often reported as pathogens, but their role in swamp eels has not been reported. The role of these bacteria in the intestines of swamp eels needs further study.

The Venn diagram (Figure 1) shows that 136 OTUs were shared between the female and male swamp eel samples, accounted for 36.66% and 79.07% of all OTUs in the two groups, respectively. The female swamp eels had more specific OTUs. The endemic species between the two groups were analyzed at the species level, which showed that the female endemic genera (>1%) comprised only Candidatus_Arthromitus. This suggested that although females had more specific OTUs, most of their relative abundances were less than 1%. *Candidatus_Arthromitus* may have protective functions on the intestines (Cox and Blaser, 2014). In this study, there were more male endemic bacteria (>1%). For example, *Lysobacter_ximonensis*, a member of this genus with strong proteolytic properties, is capable of cleaving various microorganisms (Gram-negative and Gram-positive bacteria) and nematodes (Christensen, 2005; Yassin et al., 2007), indicating that they have special biological functions in microbial ecosystems (Du et al., 2015). *Lachnospiraceae* can also synthesize butyric acid from undigested nutrients, and many extracts of *Lachnospiraceae* can be used as probiotics (Nava et al., 2010). The results showed that these endemic bacteria had specific functions. Interestingly, these endemic bacteria did not show dominant differences in the LEfSe analysis, but some symbiotic bacteria showed significant differences, indicating that these symbiotic bacteria may be sex-dependent. We therefore hypothesized that the abundance of these symbiotic bacteria might promote or delay the sex reversal process of swamp eels, which needs further study.

The pituitary gland plays a vital role in sex reversal. Increased gonadotropic hormone (GTH) during sex differentiation induces female-to-male sex reversal in the swamp eel (Liu, 1944). Estradiol can regulate intestinal flora (Benedek et al., 2017). The colonization of microorganisms increased the level of serum testosterone (Markle et al., 2013). These findings indicate a meaningful relationship between sex hormones and intestinal microorganisms. Also, the effect of exogenous hormones on the secretion of sex hormones in swamp eels is similar to that in dioecious fish. The female has mainly an increase in estradiol, and the male primarily has an increase in testosterone. The above reports prove that both sex hormones and intestinal flora play a key role in the sex reversal of swamp eels. Our hypothesis is as follows. Consider a microbiota that interacts with GTH or sex hormones in the swamp eel. When it proliferates, the amount of GTH or sex hormone secretion in the swamp eel is increased (or decreased), thereby reaching to above (or below) the threshold of induced reversal, eventually causing sex reversal to start (or stop). It is difficult to determine whether the sex reversal of swamp eels is directly mediated by microflora. Additional studies are desirable to determine the causal role of microflora in the sex reversal of swamp eels and explore the mechanisms involved.

Artificial breeding of swamp eels is a challenging task. We have conducted a long-duration experiment and achieved success after many failures. We have shown the great interestingness of this data. This study suggested that there are significant differences in the intestinal flora of male and female swamp eels. We speculated that sex reversal was partially related to the intestinal flora. These results provide useful information and a new research direction for the study of the sex reversal mechanism in swamp eels.

## Conclusion

The Alpha diversity of female swamp eels was higher than that of male swamp eels, and the species diversity of female swamp eels was also significantly higher (*p* < 0.05, Tukey’s HSD) than that of male swamp eels, as measured by the Shannon index. Beta diversity analysis showed significant differences in intestinal flora between male and female swamp eels.

The relative abundance of Actinobacteria and Spirochaetes was significantly higher (*p* < 0.05) in female swamp eels than in male swamp eels at the phylum level. The relative abundance of Mycobacterium, Bacillus, and Vagococcus was significantly higher (*p* < 0.05) in female swamp eels than in male swamp eels at the genus level.

LEfSe analysis showed that the dominant bacteria in male swamp eels were *Cetobacterium*, while those in the female swamp eels were *Clostridium_sp_ND2*, *Mycobacterium*, *Alphaproteobacteria*, and *Bacilli*.

There was a significant difference in intestinal flora between male and female swamp eels. These dramatic differences between the sexes deserve a more in-depth study on sex reversal in the future.

## Data Availability Statement

The datasets generated for this study can be found in the SRP230335.

## Ethics Statement

The animal study was reviewed and approved by the Animal care and use committee of Jiangxi Agricultural University.

## Author Contributions

QZ and JZ designed the study. YW, ZW, MG, XY, YL, ZZ, WZ, and CH collected the swamp eel samples. YW and WJ performed the experiments. YW analyzed the data. YW, JZ, and QZ wrote and revised the manuscript.

## Conflict of Interest

The authors declare that the research was conducted in the absence of any commercial or financial relationships that could be construed as a potential conflict of interest.

## Footnotes

1 http://drive5.com/uparse/

2 https://www.arb-silva.de/

## Supplementary Material

FIGURE S1 Rarefaction curve for all samples.

FIGURE S2 Circos Diagram between the MM group and the FM group on phylum level.

FIGURE S3 PLS-DA scores plot derived from the MM group and the FM group.

FIGURE S4 Species difference analysis of the MM group and the FM group at phylum level.

FIGURE S5 Species difference analysis of the MM group and the FM group at genus level.

FIGURE S6 The unique Species derived from the FM group at the species level (>1%).

FIGURE S7 The unique Species derived from the MM group at the species level (>1%).

FIGURE S8 LEfSe map of species differences between the FM group and MM group at the species level (highly significant bacteria with LDA score >2).

TABLE S1 Formulation of the experimental diets (% dry weight). ^a^These materials were provided by JiangXi for Farmer Bio-Tech Co. Ltd. (Nanchang, Jiangxi, China). ^b^Premix (in per kg diet): VA 5,000 IU, VB_1_ 25 mg, VB_2_ 45 mg, VB_6_ 20 mg, VB_12_ 0.1 mg, VK_3_ 10 mg, VE 200 mg, VC 200 mg, VD_3_ 2,500 IU, inositol 200 mg, Ca – pantothenic acid 60 mg, nicotinic acid 200 mg, folic acid 10 mg, biotin 1.5 mg, choline chloride 2,500 mg, NaSeO_3_⋅5H_2_O 0.3 mg, CoCl_2_⋅6H_2_O 0.4 mg, KI 0.8 mg, CuSO_4_⋅5H_2_O 10 mg, MnSO_4_⋅4H_2_O 20 mg, ZnSO_4_⋅H_2_O 50 mg, FeSO_4_⋅7H_2_O 150 mg, MgSO_4_⋅7H_2_O 500 mg, NaCl 1,000 mg, Ca(H_2_PO_3_)_2_ 12,450 mg.
