# Supplementary figures and images for: Analysis of the Intestinal Flora in Male Versus Female Swamp Eels (Monopterus albus)
Source: Front Microbiol. 2020 Apr 30;11:699. doi: 10.3389/fmicb.2020.00699 (PMC7203450; doi:10.3389/fmicb.2020.00699)

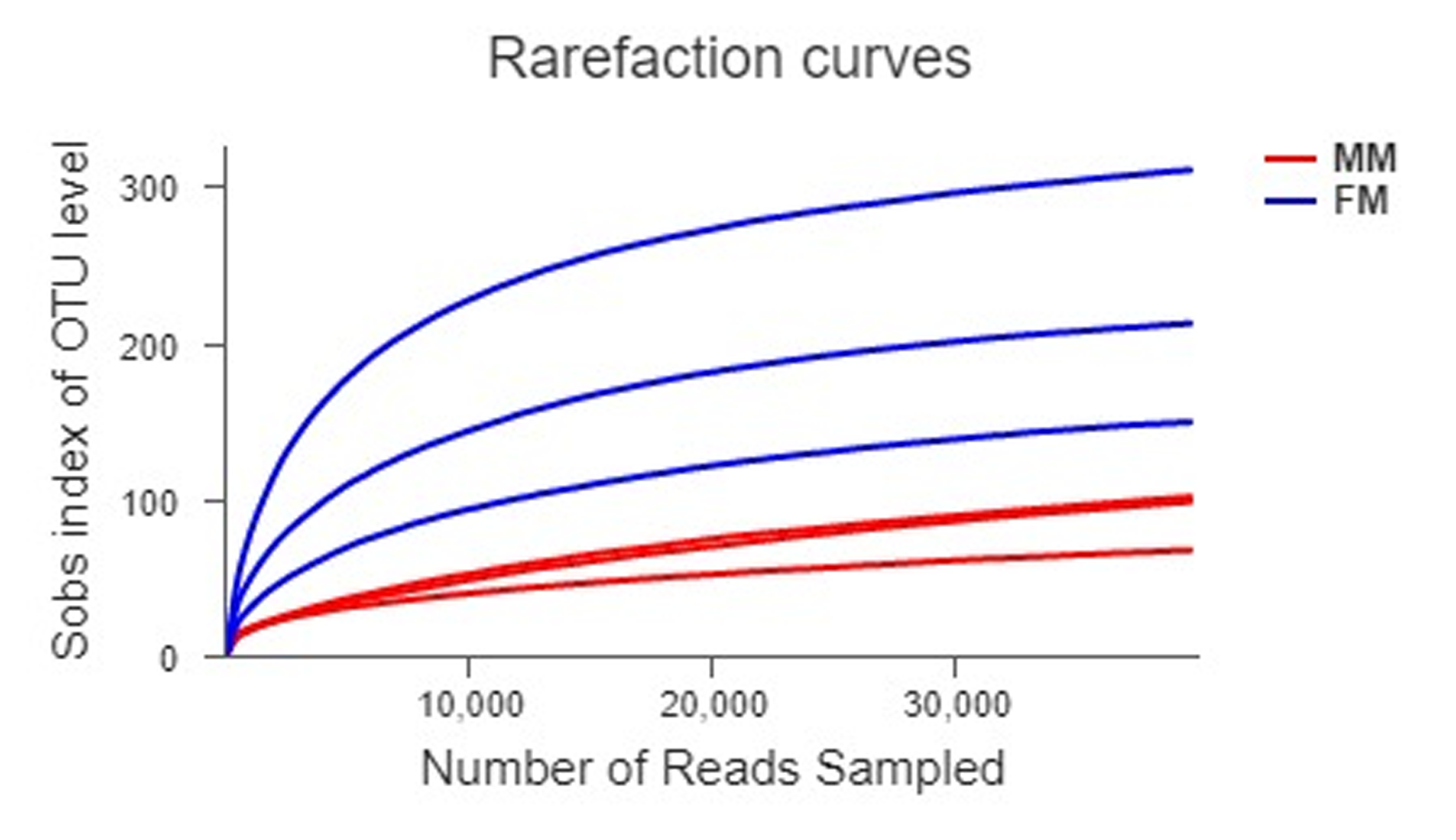

Supplement: FIGURE S1 — Rarefaction curve for all samples. [file Data_Sheet_1.zip › figureS1.tiff]

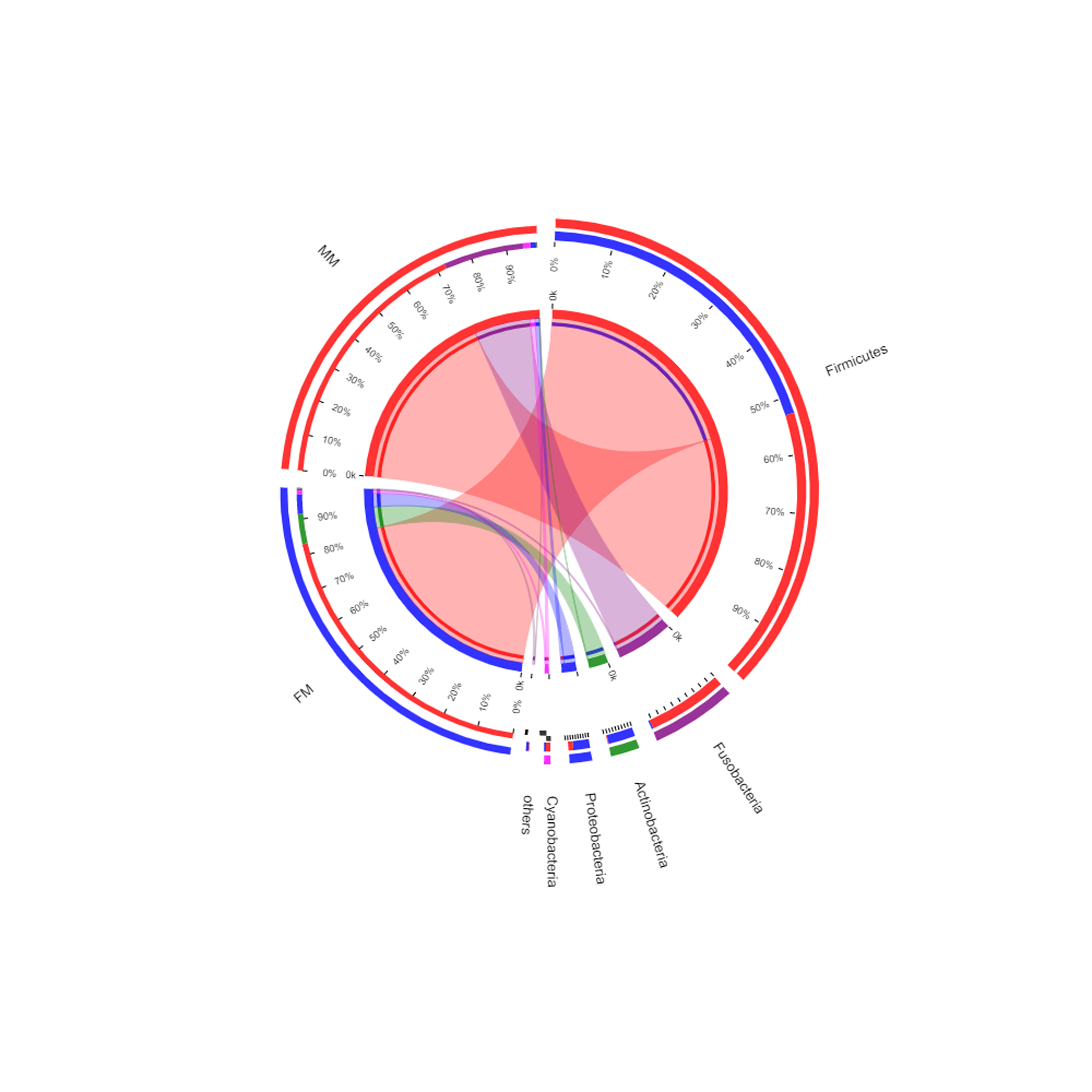

Supplement: FIGURE S1 — Rarefaction curve for all samples. [file Data_Sheet_1.zip › figureS2.tiff]

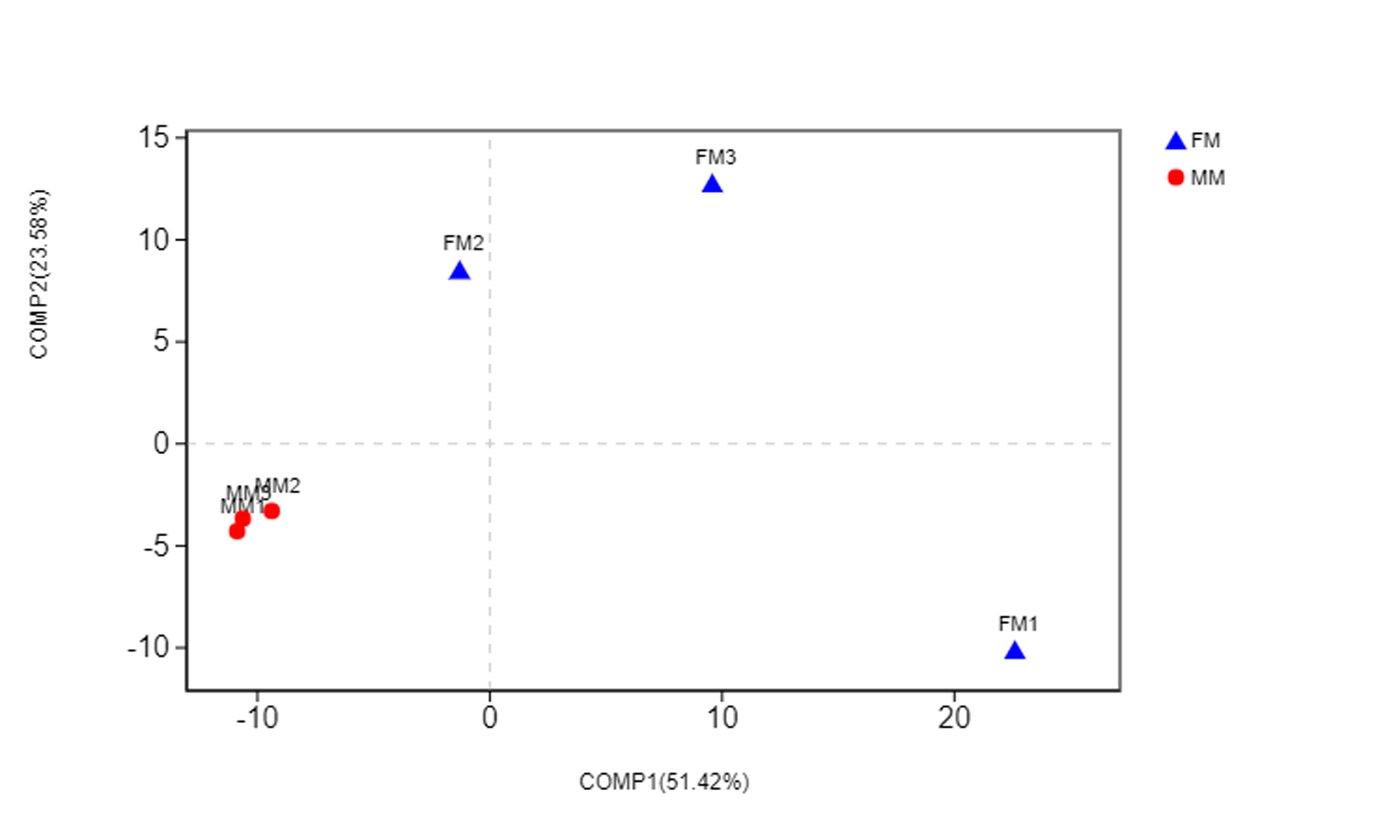

Supplement: FIGURE S1 — Rarefaction curve for all samples. [file Data_Sheet_1.zip › figureS3.tiff]

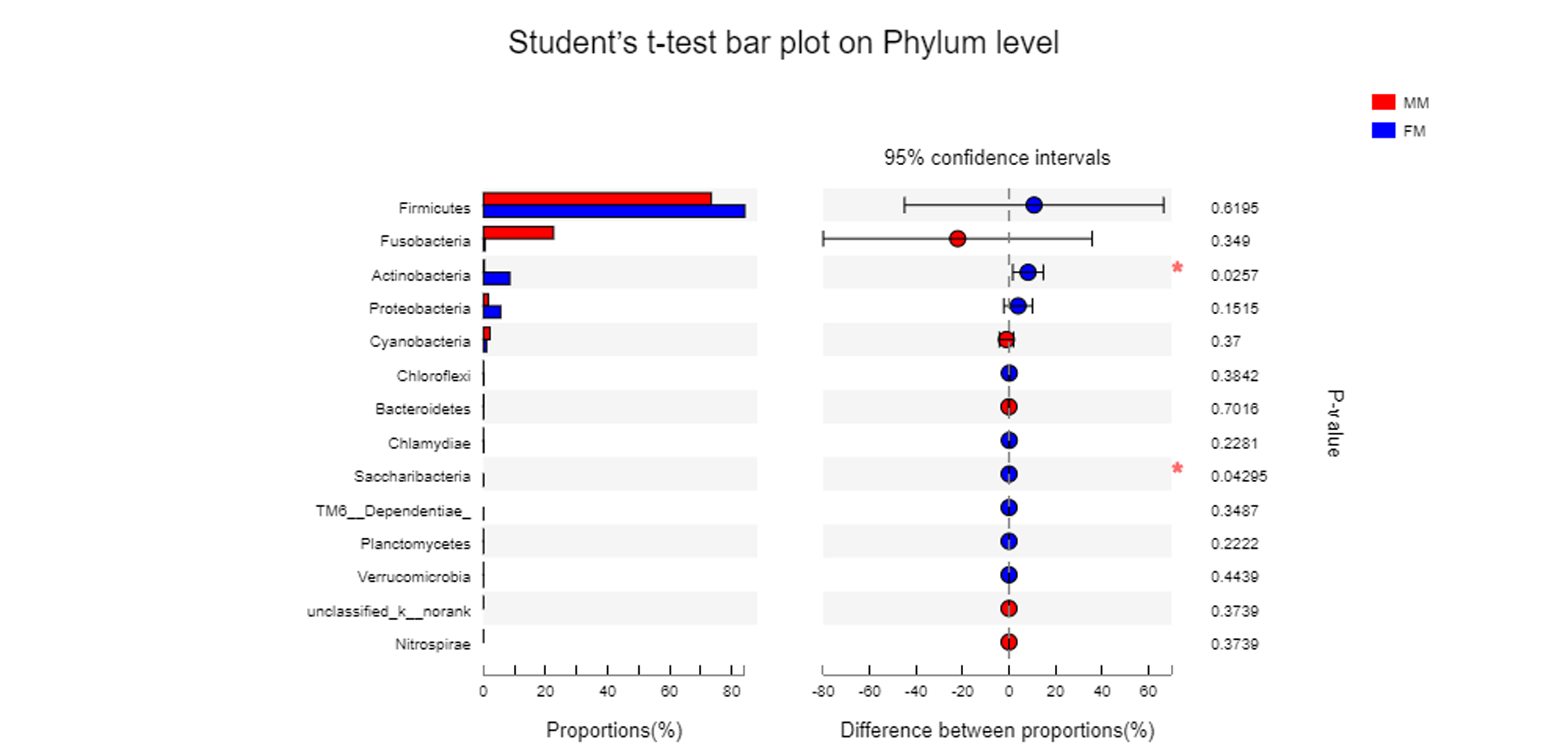

Supplement: FIGURE S1 — Rarefaction curve for all samples. [file Data_Sheet_1.zip › figureS4.tiff]

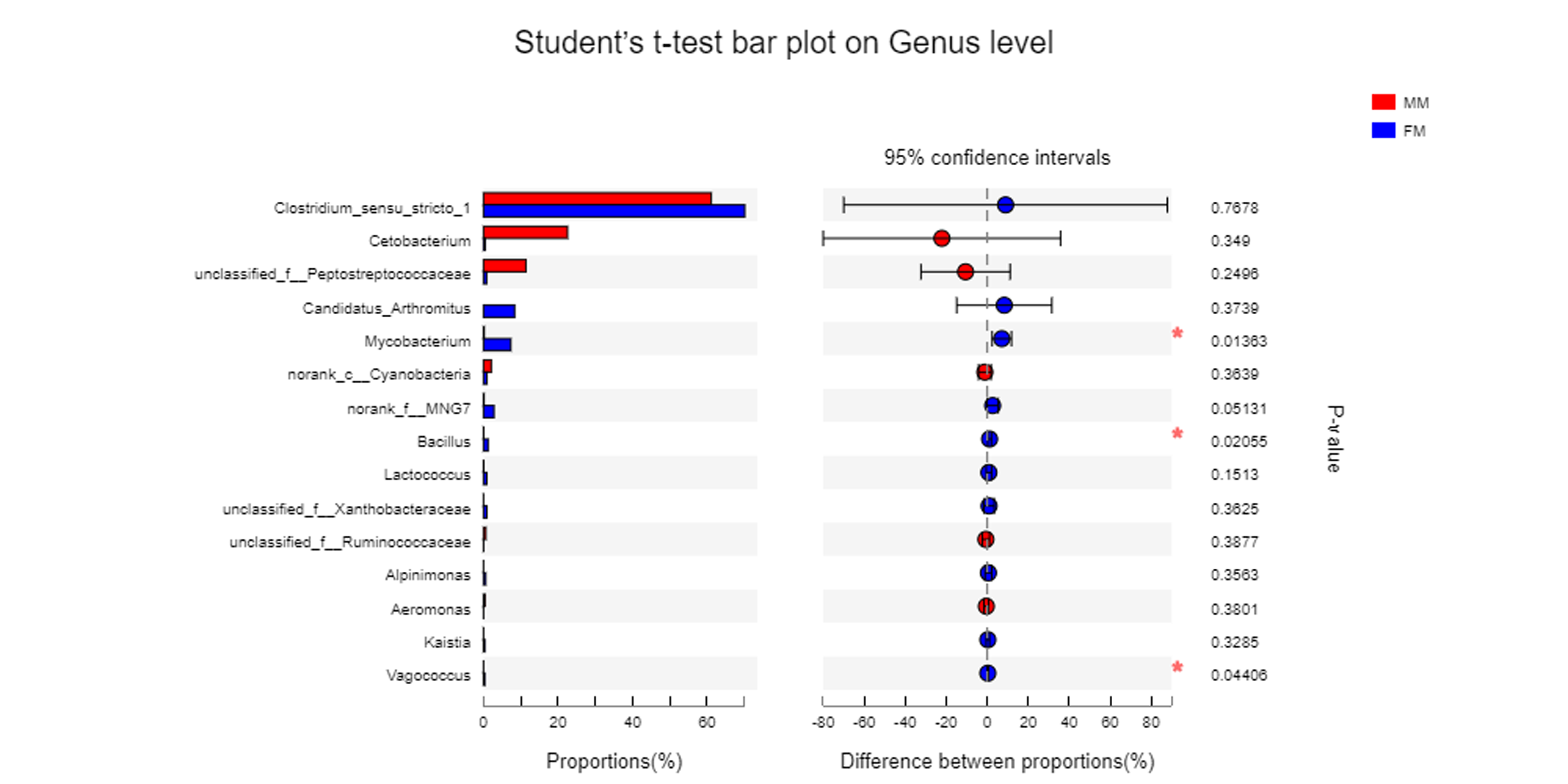

Supplement: FIGURE S1 — Rarefaction curve for all samples. [file Data_Sheet_1.zip › figureS5.tiff]

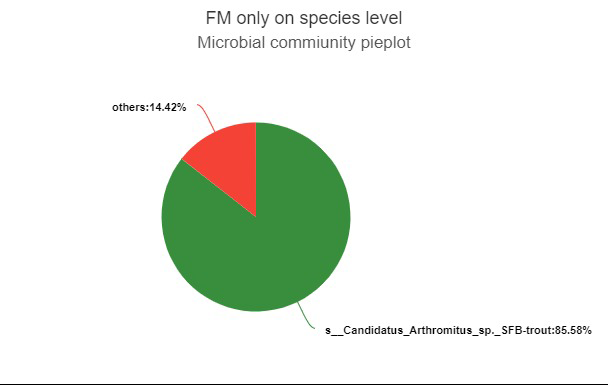

Supplement: FIGURE S1 — Rarefaction curve for all samples. [file Data_Sheet_1.zip › figureS6.tiff]

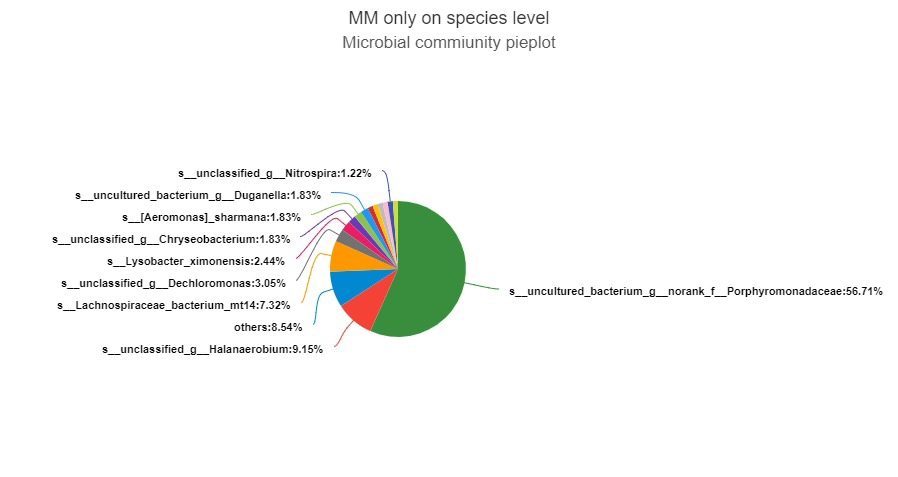

Supplement: FIGURE S1 — Rarefaction curve for all samples. [file Data_Sheet_1.zip › figureS7.tiff]

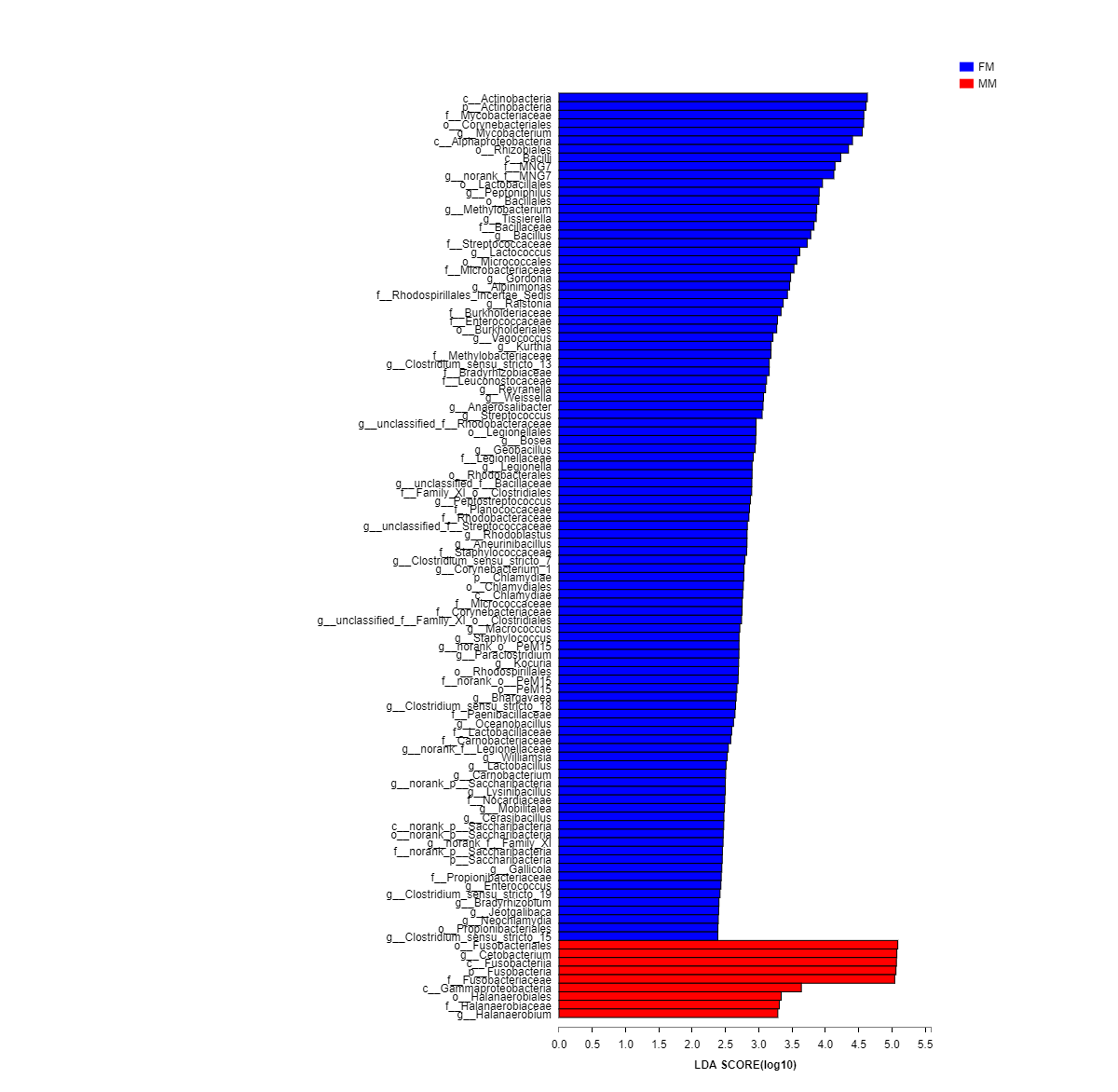

Supplement: FIGURE S1 — Rarefaction curve for all samples. [file Data_Sheet_1.zip › figureS8.tiff]
