# Supplementary material for: Analysis of the Intestinal Flora in Male Versus Female Swamp Eels (Monopterus albus)
Source: Front Microbiol. 2020 Apr 30;11:699. doi: 10.3389/fmicb.2020.00699 (PMC7203450; doi:10.3389/fmicb.2020.00699)
Supplement: TABLE S1 — Formulation of the experimental diets (% dry weight). aThese materials were provided by JiangXi for Farmer Bio-Tech Co. Ltd. (Nanchang, Jiangxi, China). bPremix (in per kg diet): VA 5,000 IU, VB1 25 mg, VB2 45 mg, VB6 20 mg, VB12 0.1 mg, VK3 10 mg, VE 200 mg, VC 200 mg, VD3 2,500 IU, inositol 200 mg, Ca – pantothenic acid 60 mg, nicotinic acid 200 mg, folic acid 10 mg, biotin 1.5 mg, choline chloride 2,500 mg, NaSeO3⋅5H2O 0.3 mg, CoCl2⋅6H2O 0.4 mg, KI 0.8 mg, CuSO4⋅5H2O 10 mg, MnSO4⋅4H2O 20 mg, ZnSO4⋅H2O 50 mg, FeSO4⋅7H2O 150 mg, MgSO4⋅7H2O 500 mg, NaCl 1,000 mg, Ca(H2PO3)2 12,450 mg. [file Data_Sheet_2.PDF]

Table S1 Formulation of the experimental diets (% dry weight).

Ingredients	Percentage
Peruvian steam fish meal[a]	55.00
Extruded soybean meal[a]	18.00
Wheat[a]	17.00
Extruded corn[a]	4.00
Soybean phosphatides[a]	2.00
Monocalcium phosphate[a]	2.00
Premix[b]	2.00
Proximate composition	
Crude protein	45.54
Crude lipid	6.96

[a] These materials were provided by JiangXi for Farmer Bio-Tech Co. Ltd. (Nanchang, Jiangxi, China).

[b] Premix (in per kg diet): VA 5 000 IU, VB_1 25 mg, VB_2 45 mg, VB_6 20 mg, VB_{12} 0.1 mg, VK_3 10 mg, VE 200 mg, VC 200 mg, VD_3 2 500 IU, inositol 200 mg, Ca—pantothenic acid 60 mg, nicotinic acid 200 mg, folic acid 10 mg, biotin 1.5 mg, choline chloride 2 500 mg, $NaSeO_3 \cdot 5H_2O$ 0.3 mg, $CoCl_2 \cdot 6H_2O$ 0.4 mg, KI 0.8 mg, $CuSO_4 \cdot 5H_2O$ 10 mg, $MnSO_4 \cdot 4H_2O$ 20 mg, $ZnSO_4 \cdot H_2O$ 50 mg, $FeSO_4 \cdot 7H_2O$ 150 mg, $MgSO_4 \cdot 7H_2O$ 500 mg, NaCl 1 000 mg, $Ca(H_2PO_3)_2$ 12 450 mg。
